# Patient Expectations, Satisfaction and Perceptions of Specialization in Family Medicine: A Cross-Sectional Study from a Health Literacy Perspective in Türkiye

**DOI:** 10.3390/healthcare14183114

**Published:** 2026-09-21

**Authors:** Egemen Tural, Seyfullah Emre Alkan

**Affiliations:** 1 Department of Family Medicine, Cerrahpaşa Faculty of Medicine, Istanbul University-Cerrahpaşa, 34098 Istanbul, Türkiye; 2 Department of Family Medicine, Cifteler Family Health Center, 26700 Eskisehir, Türkiye; s.alkan@saglik.gov.tr; 3 Department of Family Medicine, Faculty of Medicine, Osmangazi University, 26040 Eskisehir, Türkiye

**Keywords:** health literacy, primary healthcare, family practice, patient satisfaction, physician–patient relations

## Abstract

**Highlights:**

**What are the main findings?**

- A total of 60.3% of patients attending a Family Health Center had inadequate or problematic–limited health literacy (HL), which was significantly associated with their expectations, satisfaction, and perceptions of family medicine specialization.
- Patients with lower HL were more likely to hold misconceptions about the roles and authority of family medicine specialists and to view hospital-based physicians as superior to primary care physicians.

**What are the implications of the main findings?**

- Health literacy-sensitive, point-of-care communication strategies—rather than generic public-awareness campaigns—may more effectively correct misconceptions about physician specialization in primary care.
- These findings support integrating health literacy screening and tailored patient education into primary care policy in health systems with structurally complex specialization models, such as Türkiye’s.

**Abstract:**

**Background/Objectives:** Patient expectations, satisfaction, and perceptions of specialization in family medicine are critical to improving primary care, and health literacy (HL) shapes patients’ engagement with health information. This study assessed these domains and their relationship with HL using a validated scale. **Methods:** In this single-center, prospective, cross-sectional survey, 370 adult patients visiting a Family Health Center in Eskişehir, Türkiye, between March and May 2025 were randomly selected after providing informed consent under ethical approval. Expectations, satisfaction, and perceptions of family medicine specialization were assessed using a study-specific structured questionnaire, while HL was measured with the validated Turkish Health Literacy Scale-32 (THLS-32). **Results:** Participants had a mean age of 39.35 ± 12.83 years, and 51.9% were female. The mean THLS-32 score was 31.03 ± 10.94, with 60.3% being classified as having inadequate or problematic–limited HL. In unadjusted analyses, HL differed significantly by sex (*p* = 0.004), education (*p* < 0.001), marital status (*p* = 0.022), occupation (*p* = 0.001, omnibus test across groups), and medication use (*p* = 0.026) and correlated negatively with age (rho = −0.315, *p* < 0.001); in the adjusted model, sex, education, age, and worker occupation remained independently associated with HL, while marital status and medication use did not. Higher HL was associated with a lower likelihood of viewing hospital physicians as superior to primary care doctors (*p* < 0.001), fewer unnecessary antibiotic requests (*p* = 0.030), and a greater preference for comprehensive examinations (*p* = 0.001). **Conclusions:** HL was significantly associated with patient expectations, satisfaction, and perceptions of specialization in family medicine. Enhancing HL through educational interventions and patient-centered communication could improve primary care effectiveness and physician–patient relationships.

## 1. Introduction

In planning efforts aimed at improving the quality of healthcare services, incorporating patients’ perspectives and sustaining the system through feedback mechanisms has consistently been a critical component. Furthermore, the tendency of patients to evaluate the functional aspects of the services they receive, rather than solely their technical dimensions, underscores the growing importance of functional quality in healthcare [1]. One such functional dimension is the transparency of the care setting itself—specifically, whether patients can accurately identify and evaluate the training status of the physicians who treat them, a challenge that, to our knowledge, has not been directly examined in primary care systems where physicians with different levels of specialization coexist within the same clinical setting.

When effectively structured, primary healthcare services—an integral part of the healthcare system—enable individuals to access prompt, continuous, and comprehensive care. Within the framework of preventive healthcare, many health problems can be resolved before they arise, while unnecessary congestion and costs in secondary and tertiary care institutions are prevented by fulfilling a “gatekeeping” role [2,3]. Family physicians, by virtue of their training and the quality of healthcare services they provide, are among the most essential components in the delivery of primary care services. In this context, patient satisfaction with family physicians and the trust they place in them not only encourages individuals to seek primary care services as their first point of contact when in need of healthcare but also promotes adherence to medical advice and care plans [4,5,6].

Health literacy (HL), as comprehensively defined by Sorensen et al., is “linked to literacy and encompasses people’s knowledge, motivation, and competencies to access, understand, appraise, and apply health information in order to make judgments and take decisions in everyday life concerning healthcare, disease prevention, and health promotion to maintain or improve quality of life” [7].

Improved HL enables patients to perform necessary actions for their own health, understand medical brochures, instructions for prescribed medications, appointment cards, physicians’ explanations, informed consent forms, and navigate complex healthcare systems [8]. Conversely, patients with inadequate HL experience communication barriers that significantly affect their disease management and are often unable to effectively express their health conditions, leading to more frequent hospital visits [9,10,11]. Additionally, inadequate HL has been linked to diminished quality of life and increased mortality risk [12]. Despite the significant public health implications of inadequate HL and its recognition as an indispensable component of preventive healthcare [13], few studies have examined how HL relates to patients’ understanding of physician specialization in primary care. In Türkiye specifically, prior research has shown that public knowledge of the family medicine system remains limited [14], yet this and comparable studies have not examined this knowledge gap through a validated health literacy framework, nor have they explored how HL might account for variation in patients’ expectations and satisfaction regarding their family physician’s specialization status. Beyond general health information processing, patients’ expectations and satisfaction in primary care are also shaped by how well they understand who is providing their care and what that provider is qualified to do. Empirical work on these domains typically relies on context-specific, item-level assessments rather than a single universally validated instrument, since the choice of measurement approach itself can substantially affect resulting satisfaction scores—for instance, different response-scale formats have been shown to yield different psychometric properties and score distributions even for comparable content—which helps explain why patient satisfaction is commonly assessed using study- or context-specific instruments rather than a single universally standardized measure [15]. This distinction is particularly relevant in health systems where multiple tiers of primary care physicians coexist within the same practice setting, as is the case in Türkiye.

In Türkiye, family medicine specialization has been structured since 2010 through both a formal specialty training program and a contractual pathway allowing practicing general practitioners to become specialists while continuing their duties [16]. As a result, Family Health Centers may be staffed concurrently by general practitioners, fully trained specialists, and contractual specialist trainees, a structural heterogeneity that can make it difficult for patients to accurately identify their physician’s qualifications.

This study aimed to assess patients’ responses to a set of study-specific items on expectations, satisfaction, and perceptions of family medicine specialization and to examine their association with health literacy among patients attending a primary care Family Health Center in Türkiye.

## 2. Materials and Methods

### 2.1. Study Design and Participants

This study was conducted at a single Family Health Center in Çifteler, a rural district of Eskişehir Province, Türkiye, where two physicians—one family medicine specialist and one general practitioner—provided care and from whose patient data were collected. For this single-center, prospective, cross-sectional survey study, participants were selected using a convenience sampling method from individuals visiting the Family Medicine Outpatient Clinic of a Family Health Center who met the study’s inclusion criteria. Patients were approached consecutively in the clinic waiting area during the study period by trained research staff; the exact number of patients approached and the number who declined participation were not systematically logged during data collection.

### 2.2. Ethical Approval and Study Process

Ethical approval was obtained on 20 February 2025 from the Scientific Research Ethics Committee of the City Hospital (Decision No: 2025/121). This study complies with the Declaration of Helsinki, adheres to the principles of Good Clinical Practice, and does not contravene ethical standards for research involving human subjects. All participants voluntarily agreed to participate and provided written informed consent prior to enrollment.

### 2.3. Sample Size Estimation

On average, 3120 patients aged 18 years and older visit the Family Health Center monthly. Over the two-month study period, this corresponded to approximately 6240 patient visits; because this figure reflects visits rather than unique individuals, it likely overestimates the true number of distinct patients in the target population and was therefore used as a conservative, upper-bound denominator for the sample size calculation. Assuming an expected proportion of 50% (to maximize the required sample size in the absence of prior data), a ±0.05 margin of error, and a 95% confidence level (Z = 1.96), the minimum sample size required for adequate precision of the proportion estimate was calculated as 362, using Cochran’s formula for finite populations. The study was ultimately completed with 370 participants.

### 2.4. Inclusion and Exclusion Criteria

Individuals were included if they voluntarily agreed to participate, were aged 18 years or older, and were cognitively capable of communication (e.g., without a diagnosis of dementia, intellectual disability, or psychosis that would impair comprehension of the questionnaire). Individuals were excluded if they declined to participate, were under 18 years of age, had a cognitive or psychiatric condition impairing comprehension of the questionnaire, or left the questionnaire substantially incomplete. Participants who responded “don’t know” to more than 20% of the THLS-32 items were also excluded, as an insufficient number of valid item responses precluded reliable index calculation. Participants with incomplete forms were not included in the final analytic sample of 370.

### 2.5. Data Collection Procedure

Following ethical approval, data were collected between 15 March 2025 and 15 May 2025. Participants were informed about the study, and those who volunteered and met inclusion criteria were administered a data collection form developed by the researchers based on a literature review. The form included questions on age, gender, marital status, income level, education level, chronic diseases, regular medication use, patient satisfaction, expectations from physicians, and perceptions and knowledge regarding family medicine specialization. The questionnaire was developed specifically for this study based on the aims and research questions. The questionnaire items concerning patient expectations, satisfaction, and perceptions of family medicine specialization were generated based on a review of the relevant literature on patient satisfaction and expectations in Turkish primary care [17,18] and the authors’ clinical experience. As this instrument was not subjected to formal psychometric validation (e.g., content validity assessment, pilot testing, exploratory factor analysis, and internal consistency testing), its use should be considered a methodological limitation, discussed further in Section 4.5. The 18 items were grouped, based on their content, into three domains: perceptions/knowledge of the family medicine specialization system (5 items), expectations regarding family physician care (8 items), and satisfaction-related beliefs and behaviors (4 items); one item reflecting a general health belief was not classified under a specific domain. These domain groupings reflect a post hoc, content-based categorization rather than empirically derived subscales; each item was therefore analyzed individually rather than as part of a composite score. An English translation of the study-developed questionnaire, comprising items on patient expectations, satisfaction, and perceptions of family medicine specialization, is provided as Supplementary Material S1 (Statements 1–18); item groupings by domain are indicated therein. Participants completed the sociodemographic, expectations, satisfaction, and perceptions sections of the questionnaire independently. The Turkish Health Literacy Scale-32 (THLS-32) [19] is a previously validated, published instrument; its full item set is therefore not reproduced here, but it is available in the original source [19]. The THLS-32 was subsequently administered face to face, with a member of the research team reading each item aloud and recording participants’ responses, in order to minimize the impact of literacy or comprehension difficulties on scale completion.

### 2.6. Scale Description and Assessment

The THLS-32, used to determine participants’ HL levels, was developed and validated by Okyay et al. in 2016 [19]; an English translation is provided as Supplementary Material S2. It consists of 32 items, each rated on a 4-point scale (1 = very easy, 2 = easy, 3 = difficult, and 4 = very difficult), plus a “don’t know” option. Prior to index calculation, raw item codes were reverse-scored (1 ↔ 4, 2 ↔ 3), so that higher values reflected greater ease, consistent with the scale’s standard scoring procedure [19]. As in the European Health Literacy Survey (HLS-EU) study [20], scale scores were then standardized to a 0–50 range using the formula: index = (arithmetic mean − 1) × [50/3], where the mean reflects the average of the reverse-scored item responses. The resulting index was categorized as follows: 0–25 = inadequate HL, >25–33 = problematic–limited HL, >33–42 = sufficient HL, and >42–50 = excellent HL. “Don’t know” responses were treated as missing and excluded from the calculation of each participant’s mean item score (see Inclusion and Exclusion Criteria for the applied threshold). The Cronbach’s alpha coefficient of the scale was 0.927 in the original validation study [19].

### 2.7. Statistical Analyses

Frequencies (*n*) and percentages (%) were used to describe demographic characteristics. The normality of continuous variables was assessed graphically and with the Shapiro–Wilk test. Median (Minimum–Maximum) values were reported for descriptive statistics.

To compare THLS-32 scores across categorical variables with more than two groups (e.g., education level, marital status, occupation, smoking, and alcohol use), the Kruskal–Wallis non-parametric variance analysis was applied. Pairwise comparisons were performed with Bonferroni correction. For dichotomous variables (e.g., gender, chronic disease status, and regular medication use), the Mann–Whitney U test was employed. Spearman’s non-parametric correlation coefficient was used to assess the relationship between age and THLS-32 scores.

A generalized linear model (GLM) with a normal (Gaussian) distribution and an identity link function was applied, with THLS-32 score as the dependent variable and gender, education level, occupation, marital status, regular medication use, and age as independent variables. Covariates were selected a priori based on variables showing statistically significant unadjusted associations with THLS-32 score in Table 1, together with age, which is a recognized correlate of health literacy in the broader literature. The associations between individual questionnaire items and THLS-32 score presented in Table 2 reflect unadjusted, exploratory comparisons and should not be interpreted as evidence of independent associations after accounting for demographic factors. These comparisons were not corrected for multiple testing beyond the Bonferroni-adjusted pairwise comparisons following significant Kruskal–Wallis tests and should therefore be considered hypothesis-generating rather than confirmatory. Statistical analyses were performed using IBM SPSS Statistics 26.0 (IBM Corp., Armonk, NY, USA). MS Excel 2024 was used solely for data organization and table formatting. A *p*-value <0.05 was considered statistically significant.

## 3. Results

The mean age of participants was 39.35 ± 12.83 years. Of the participants, 51.9% (*n* = 192) were female, and 48.1% (*n* = 178) were male. Regarding education level, 17.0% (*n* = 63) were primary school graduates, 4.9% (*n* = 18) completed elementary/middle school, 36.5% (*n* = 135) were high school graduates, 8.4% (*n* = 31) were vocational school graduates, 32.1% (*n* = 119) were university graduates, and 1.1% (*n* = 4) were literate without formal schooling.

The mean THLS-32 score of participants was 31.03 ± 10.94. According to the THLS-32 classification, 27.3% (*n* = 101) were categorized as having inadequate HL, 33.0% *(n* = 122) as having problematic–limited HL, 20.2% (*n* = 75) as having sufficient HL, and 19.5% (*n* = 72) as having excellent HL.

A weak, negative, and statistically significant correlation was found between age and THLS-32 scores (rho = –0.315; *p* < 0.001). The comparison of THLS-32 scores according to the participants’ demographic characteristics is presented in Table 1.

Regarding perceptions and expectations related to family medicine specialization, participants with higher HL were significantly less likely to consider physicians working in hospitals superior to those in Family Health Centers (*p* < 0.001). They were also less likely to state that they would be pleased if their physician did not refuse an antibiotic request (*p* = 0.030), and more likely to prefer a comprehensive examination over a complaint-focused visit at each clinic attendance (*p* = 0.001). Full item-level comparisons for all statements are presented in Table 2.

According to the results of the GLM, in which the THLS-32 score was the dependent variable and sex, education level, marital status, occupation, regular medication use, and age were the independent variables, a one-unit increase in age resulted in a −0.196-unit change in the THLS-32 score. Regarding the education level, compared with illiterate/primary/secondary school graduates, high school graduates had scores 7.066 points higher, vocational school graduates 10.005 points higher, and university graduates 9.215 points higher. Additionally, participants who were workers had a mean THLS-32 score 3.961 points lower than those who were unemployed/housewives. Although marital status showed a significant unadjusted association with the THLS-32 score (Table 1), it was not significantly associated with HL after adjustment for the other covariates (Table 3), suggesting that its unadjusted association was largely confounded by age.

## 4. Discussion

This study examined patient expectations, satisfaction, and perceptions of family medicine specialization among primary care attendees, framed within the construct of health literacy (HL). Consistent with Sorensen et al.’s conceptualization of HL as the capacity to access, understand, appraise, and apply health information [7], our finding that 60.3% of participants demonstrated inadequate or problematic–limited HL suggests that a majority of patients in this setting may lack the competencies needed to fully engage with and critically evaluate the primary care services available to them—including distinguishing between the qualifications of the physicians who provide that care. This prevalence is broadly consistent with reports from the European Health Literacy Survey [20] and other national studies, indicating that limited HL is not an isolated local phenomenon but a structural challenge shared across health systems, one with direct implications for how patients navigate increasingly differentiated primary care models such as Türkiye’s.

Beyond the sociodemographic correlates of HL identified in unadjusted analyses—including educational attainment, sex, marital status, occupation, and age—sex, education, occupation (specifically, being a worker), and age remained independently associated with HL after adjustment, whereas marital status and regular medication use did not, suggesting that their unadjusted associations were largely confounded by age. Beyond its association with patient expectations, satisfaction, and perceptions in this study, HL has broader downstream implications; individuals with higher HL generally demonstrate better self-management of chronic conditions, and a recent systematic review of 23 studies found that higher HL was associated with lower healthcare costs across several cost categories—including medical, inpatient, and emergency care costs—although the strength of evidence varied and the association’s causal nature remains unclear [21]. A more clinically relevant finding was the association between HL and patients’ perceptions of family medicine. Participants with lower HL were more likely to consider hospital-based physicians superior to those in primary care, and a considerable proportion exhibited misconceptions about the scope and authority of family medicine specialists. This pattern suggests that inadequate HL is associated not only with limited engagement with health information in the abstract but also with how patients perceive, trust, and describe their preferences between different tiers of the healthcare system; given the cross-sectional design, whether these associations translate into actual care-seeking behavior or affect the functioning of primary care as a gatekeeping mechanism cannot be determined from this study and would require prospective research.

### 4.1. Factors Influencing Health Literacy and Clinical Implications

Improving HL has the potential to enhance patient health status, disease prevention, and disease management [22]. A study by Altin and Stock in Germany demonstrated that individuals with adequate subjective HL who engaged in more patient-centered communication and shared decision making with their general practitioners were more likely to report satisfaction with their care [23]. Similarly, Mefford et al. [24] in the United States found that limited HL, assessed through a three-item instrument, was associated with reduced treatment satisfaction among patients receiving venous thromboembolism therapy. These findings highlight that while brief HL screening tools may be convenient, their limited scope could compromise measurement reliability. Using validated, multidimensional instruments is therefore preferable for robust HL assessment.

HL is a complex construct shaped by various individual and contextual factors. In a cross-sectional study involving 399 participants in primary care, Šulinskaite et al. reported that inadequate and problematic HL were predominant, whereas sufficient and excellent HL were more common among younger individuals, students, those living with a partner, and participants with higher educational levels; gender was not a significant determinant of HL [25]. Shahid et al. [26], using the Test of Functional Health Literacy in Adults, similarly demonstrated that older adults exhibited significantly lower HL, and over half of the participants with secondary or lower education had inadequate HL.

In a study conducted in Türkiye by Başaran and Altınel involving 306 participants, health literacy was assessed using the same THLS-32 instrument employed in our study. The research study, which examined the relationship between HL and rational drug use among patients presenting to the emergency department, reported a mean THLS-32 score of 32.08 ± 8.70 points [27]. Consistent with our findings, higher education and not using regular medication were associated with significantly higher HL, whereas an increase in age correlated negatively with HL. In our cohort, HL was notably higher among women, individuals with at least secondary education, single participants, civil servants or students, and those not on chronic medication. The gender disparity may reflect greater proactivity of women in seeking health information [28], while the education-related difference may stem from enhanced cognitive and functional skills to access and interpret health information; neither explanation was directly tested in the present study. Similarly, the elevated scores in civil servants and students may be attributed to greater educational exposure and easier access to information. Conversely, lower HL observed in older adults and individuals on long-term medication could be related to higher chronic disease burden, treatment complexity, cognitive slowing, and memory impairments. These findings underscore the importance of targeted HL interventions for vulnerable groups, particularly those with lower educational attainment, older adults, and patients with chronic conditions. In practice, this could involve routinely offering simplified, written after-visit summaries to patients aged 65 and older or those on multiple chronic medications, training family physicians in the teach-back technique to verify patient understanding before the end of a visit, and prioritizing plain-language explanations over medical terminology when discussing chronic disease management with patients with lower educational attainment.

### 4.2. Patient Satisfaction and Physician–Patient Relationship

Physician–patient relationship quality and continuity of care are well-established correlates of patient satisfaction in primary care. In a U.S. family medicine clinic, longer waiting times and inconsistent physician continuity were linked to lower satisfaction [29], while a Turkish study found that higher electronic HL improved treatment adherence within the physician–patient relationship [30]. Being seen by a specific, consistent physician and having shorter distances to facilities have similarly been linked to higher satisfaction [31]; notably, one study found that rural patients, despite facing greater access barriers, more often reported being treated with dignity and respect than urban patients [32]. While these structural factors were not directly assessed here, our findings extend the literature by examining how HL relates to patients’ attitudes toward two concrete aspects of care: antibiotic-seeking behavior and preference for comprehensive versus complaint-focused examinations.

In our study, the physician–patient relationship, patient preferences, and satisfaction were assessed through several statements. A statistically significant difference in THLS-32 scores was found for responses to the statement *“I would be pleased if my family physician does not refuse my request for antibiotics.”* Those who responded “Disagree” had significantly higher THLS-32 scores compared with those who answered otherwise. This finding is clinically relevant, as patient demand is a well-documented driver of inappropriate antibiotic prescribing in primary care, with physicians often prescribing against their own clinical judgment to meet such expectations [33]; health literacy-sensitive counselling on when antibiotics are not indicated may therefore represent a targetable point for antimicrobial stewardship.

A U.S. study found that patients were generally open to comprehensive physical examinations, including additional maneuvers beyond the presenting complaint, and believed that this could strengthen the therapeutic relationship even when performed routinely rather than only when symptomatic [34].

In our study, participants who disagreed with the following statement had significantly higher mean THLS-32 scores than those who agreed or were undecided: *“When visiting a family health center for an examination, I prefer to be examined only for the current complaint rather than having a full general examination.”*

These findings suggest that physician–patient communication and patients’ attitudes toward clinical encounters may be relevant to patient satisfaction and treatment adherence, consistent with the broader literature reviewed above. In our sample, participants with higher HL expressed less willingness to request antibiotics against their physician’s judgment and expressed a stronger preference for comprehensive over complaint-focused examinations; as these were self-reported attitudes rather than observed behavior or clinical outcomes, they should be interpreted as expressed preferences rather than evidence of actual prescribing requests or improved trust. Whether patient-centered approaches emphasizing comprehensive examinations and communication strategies translate into improved satisfaction or health outcomes in this population would need to be tested directly in future research.

### 4.3. Perceptions of Family Medicine Specialization and Its Relationship with Health Literacy

As previously noted, the multi-tiered structure of family medicine service delivery in Türkiye may itself contribute to patients’ difficulty in accurately understanding the roles and qualifications of their physicians. In a study conducted in Türkiye by Özdemir et al. with 489 participants which evaluated the knowledge of individuals who were less inclined to utilize family medicine services, it was found that the overall knowledge level about the family medicine system was still insufficient. The study suggested that physicians should offer screenings and conduct periodic health examinations and that public-awareness campaigns about family medicine practices could be effectively disseminated through media channels [14].

In our study, a statistically significant difference in THLS-32 scores was observed based on responses to the statement *“In Family Health Centers, only general practitioners (non-specialists) work as physicians.”* Additionally, participants who agreed with the following statements had significantly higher THLS-32 scores compared with those who disagreed or were undecided: *“I believe I have sufficient knowledge about the authorities and competencies of family medicine specialists.” “A family medicine specialist has the authority to issue medication reports for chronic diseases.”* Conversely, participants who disagreed with the following statement scored significantly higher than those who agreed or were undecided: *“Physicians working in hospitals are superior to those working in family health centers.”*

These findings highlight the presence of informational gaps in the community regarding family medicine services and specialization. In our sample, higher health literacy was associated with more accurate perceptions of family medicine practices. This relationship between HL and perception accuracy is consistent with the broader literature on how HL shapes the patient–physician relationship. Health literacy is a recognized determinant of the quality of patient–physician communication, encompassing patients’ ability to access, understand, and act on health-related information, as well as their engagement in question asking during clinical encounters—a process frequently constrained by time pressures in primary care [35]. Separately, health education and literacy have been identified as a modifiable, patient-centered contributor to trust in physicians, with higher health literacy being associated with stronger patient–physician trust and lower literacy being identified as a barrier to it [36]. Together, limited health literacy’s effects on communication and trust offer a plausible, literature-supported explanation for why patients with lower HL in our sample were more likely to hold inaccurate perceptions of family medicine specialization. One possible explanation is that these misconceptions persist because the coexistence of general practitioners, contractual residents, and specialists within the same centers is rarely made explicit to patients; a broader cultural tendency in Türkiye to associate hospital-based care with higher technical competence may also contribute, although this mechanism was not directly assessed in the present study. Practically, such misperceptions could plausibly contribute to unnecessary self-referral to secondary or tertiary facilities for problems manageable in primary care, potentially straining the gatekeeping function family medicine is meant to serve; however, this study did not directly assess referral behavior, and this remains a hypothesis for future research. Rather than relying solely on generic public-awareness campaigns, more targeted measures—such as clearly displaying physicians’ titles and specialization status at the point of care and having physicians proactively clarify their qualifications during consultations—may more directly address the specific knowledge gaps identified here.

### 4.4. Clinical and Research Implications

Given the cross-sectional, single-center design and the use of a study-specific, non-validated questionnaire, these findings should be interpreted as associations rather than evidence of causal or intervention effects. They suggest, as a hypothesis for future research, that targeted communication strategies—such as clearly displaying physicians’ titles and specialization status at the point of care, and training physicians in the teach-back technique—may be worth evaluating for their impact on patient understanding and satisfaction. Multi-center studies using validated instruments and adjusted, multivariable analyses are needed to confirm the generalizability of these findings and to formally test the effectiveness of such interventions before they are adopted in policy or practice. Future research could also examine the role of nurse–patient interactions in shaping patients’ perceptions of their physicians and of primary care practice more broadly.

### 4.5. Study Limitations

This study has several limitations. First, its cross-sectional design precludes establishing causality between HL and patient satisfaction or perceptions. Second, the questionnaire employed a mixed administration format—self-completion for the sociodemographic, expectations, satisfaction, and perceptions items and face-to-face administration for the THLS-32—which may have introduced differential response bias across sections, including social desirability bias in the face-to-face component and possible misinterpretation of items in the self-completed component. Third, this was a single-center study conducted among patients actively attending a Family Health Center, which may introduce selection bias: individuals who seek primary care may differ systematically in HL, health-seeking behavior, and engagement with the healthcare system compared with the general community, including those who avoid or delay care. This could plausibly bias our HL estimates and the observed associations with perceptions of specialization in either direction, and limits generalizability beyond care-seeking populations in similar geographic and institutional contexts. In particular, patients with higher socioeconomic status may be more likely to bypass public family medicine services in favor of private healthcare institutions in Türkiye; consequently, our sample may underrepresent this group, and the relationship between socioeconomic status and HL in populations who preferentially use private care remains an open question for future research. Furthermore, because the number of patients approached and the number of those who declined participation were not systematically recorded during data collection, the response rate could not be calculated, limiting our ability to formally assess the risk of non-response bias. Moreover, the item-level associations reported in Table 2 were based on unadjusted, exploratory analyses without correction for multiple comparisons beyond pairwise post hoc tests and should be interpreted as hypothesis-generating rather than as evidence of independent associations. Additionally, the absence of a standardized, validated patient satisfaction instrument is another limitation. Lastly, because item-level THLS-32 responses were not retained, the internal consistency of the scale could not be verified within this specific sample.

## 5. Conclusions

This study found that HL was significantly associated with patients’ expectations, satisfaction, and perceptions of family medicine specialization in a single primary care setting in Türkiye, with 60.3% of participants exhibiting inadequate or problematic–limited HL. Patients with higher HL held more accurate perceptions of family medicine specialists’ roles and authority and expressed different attitudes toward antibiotic use and examination preferences, while substantial misconceptions persisted among those with lower HL, particularly regarding the distinctions among general practitioners, contractual residents, and specialists.

## Figures and Tables

**Table 1:** Comparison of Turkish Health Literacy Scale-32 (THLS-32) scores according to participants’ demographic characteristics.

			THLS-32 SCORE	Test Statistic	
		*n* (%)	Median (Min–Max)	z; χ^2^	*p*
Gender	Female	192 (51.9)	32.0 (0.0–50.0)	z = 2.843	**0.004**
	Male	178 (48.1)	29.2 (0.0–50.0)		
Education Level	Illiterate/primary school/middle school ^abc^	85 (23.0)	23.9 (0.0–50.0)	χ^2^ = 50.059	**<0.001**
	High school ^a^	135 (36.5)	30.7 (0.0–50.0)		
	Vocational school ^b^	31 (8.4)	34.4 (0.0–50.0)		
	University ^c^	119 (32.1)	33.3 (13.0–50.0)		
Marital Status	Single ^a^	101 (27.3)	32.8 (5.2–50.0)	χ^2^ = 7.618	**0.022**
	Married ^a^	246 (66.5)	29.4 (0.0–50.0)		
	Widowed/divorced	23 (6.2)	29.7 (10.9–50.0)		
Occupation	Unemployed/housewife	99 (26.7)	30.7 (0.0–50.0)	χ^2^ = 19.633	**0.001**
	Civil servant ^ab^	92 (24.9)	32.0 (13.0–50.0)		
	Worker ^a^	49 (13.2)	28.1 (0.0–48.4)		
	Self-employed	92 (24.9)	30.7 (0.0–50.0)		
	Retired ^b^	18 (4.9)	22.4 (13.5–40.1)		
	Student	20 (5.4)	33.1 (16.7–50.0)		
Income Level	Below minimum wage	110 (29.7)	30.21 (0.00–50.00)	χ^2^ = 7.441	0.059
	Minimum wage	109 (29.5)	29.17 (0.00–50.00)		
	Twice the minimum wage	111 (30.0)	31.77 (0.00–50.00)		
	Three times or more the minimum wage	40 (10.8)	31.75 (0.00–50.00)		
Chronic Disease	None	285 (77.0)	30.7 (0.0–50.0)	z = 1.687	0.092
	Present	85 (23.0)	28.6 (0.0–50.0)		
Regular Medication Use	None	290 (78.4)	31.2 (0.0–50.0)	z = 2.223	**0.026**
	Present	80 (21.6)	28.2 (0.0–50.0)		
Smoking Status	No	216 (58.4)	31.2 (0.0–50.0)	χ^2^ = 2.375	0.305
	Yes	126 (34.1)	29.7 (0.0–50.0)		
	Quit	28 (7.6)	30.9 (11.5–50.0)		
Alcohol Consumption	No	300 (81.1)	31.2 (0.0–50.0)	χ^2^ = 3.924	0.141
	Yes	59 (15.9)	29.7 (0.0–47.9)		
	Quit	11 (3.0)	25.5 (18.7–46.9)		

z: Mann–Whitney U test, χ^2^: Kruskal–Wallis test, ^abc^: Same letters express significant differences between groups. Bold indicates statistically significant values (*p* < 0.05).

**Table 2:** Comparison of Turkish Health Literacy Scale-32 (THLS-32) scores based on responses to statements related to family medicine.

	THLS-32 SCORE				
	Disagree	Agree	Undecided	Test Statistic	
	*n* (%) Median (Min–Max)	*n* (%) Median (Min–Max)	*n* (%) Median (Min–Max)	χ^2^	*p*
“In Family Health Centers, only general practitioners (non-specialists) work as physicians.”	209 (56.5)	85 (23.0)	76 (20.5)	χ^2^ = 9.001	**0.011**
	31.8 (0.0–50.0) ^a^	29.2 (0.0–50.0)	29.6 (0.0–50.0) ^a^		
“A family medicine specialist becomes a specialist after one year of training.”	128 (34.6)	120 (32.4)	122 (33.0)	χ^2^ = 3.279	0.194
	29.4 (0.0–50.0)	32.0 (0.0–50.0)	30.2 (0.0–50.0)		
“It is important to me that my family physician is a family medicine specialist.”	40 (10.8)	297 (80.3)	33 (8.9)	χ^2^ = 3.072	0.215
	33.8 (0.0–49.5)	30.2 (0.0–50.0)	30.2 (0.0–50.0)		
“I believe I have sufficient knowledge about the authorities and competencies of family medicine specialists.”	85 (23.0)	197 (53.2)	88 (23.8)	χ^2^ = 8.943	**0.011**
	29.2 (0.0–50.0) ^a^	32.3 (0.0–50.0) ^a^	30.2 (0.0–50.0)		
“A family medicine specialist has the authority to issue medication reports for chronic diseases.”	72 (19.5)	223 (60.3)	75 (20.2)	χ^2^ = 6.579	**0.037**
	29.7 (0.0–50.0)	31.2 (0.0–50.0) ^a^	29.2 (0.0–48.9) ^a^		
“If I had to change my family physician one day, I would prefer a doctor who fulfills my requests most easily rather than the one I think is the most knowledgeable or skilled.”	249 (67.3)	94 (25.4)	27 (7.3)	χ^2^ = 8.762	**0.013**
	30.6 (0.0–50.0)	33.1 (0.0–50.0) ^a^	28.6 (0.0–37.5) ^a^		
“If I believe I will recover with antibiotics, I would not hesitate to tell my physician.”	96 (25.9)	242 (65.4)	32 (8.7)	χ^2^ = 1.319	0.517
	32.0 (0.0–50.0)	30.2 (0.0–50.0)	29.9 (0.0–49.5)		
“I would be pleased if my family physician does not refuse my request for antibiotics.”	150 (40.5)	171 (46.2)	49 (13.3)	χ^2^ = 6.981	**0.030**
	31.5 (0.0–50.0) ^ab^	30.2 (0.0–50.0) ^a^	28.7 (0.0–50.0) ^b^		
“I am pleased if my family physician closely follows medical advancements.”	17 (4.6)	346 (93.5)	7 (1.9)	χ^2^ = 2.141	0.343
	34.4 (0.0–50.0)	30.5 (0.0–50.0)	29.2 (0.0–39.1)		
“I would take a medication prescribed by my family physician without hesitation.”	43 (11.7)	288 (77.8)	39 (10.5)	χ^2^ = 1.482	0.477
	29.7 (0.0–50.0)	30.7 (0.0–50.0)	28.1 (0.0–48.9)		
“Except for emergencies, I would not consider going to a hospital for health problems without my family physician’s referral.”	117 (31.6)	197 (53.2)	56 (15.2)	χ^2^ = 0.077	0.962
	30.7 (0.0–50.0)	30.2 (0.0–50.0)	30.2 (0.0–48.9)		
“I prefer to be able to go directly to a training and research hospital, city hospital, or medical faculty hospital whenever I want.”	140 (37.8)	180 (48.7)	50 (13.5)	χ^2^ = 0.061	0.970
	30.2 (0.0–50.0)	30.9 (0.0–50.0)	29.7 (0.0–50.0)		
“Physicians working in hospitals are superior to those working in family health centers.”	236 (63.8)	74 (20.0)	60 (16.2)	χ^2^ = 22.065	**<0.001**
	32.8 (0.0–50.0) ^a^	26.8 (0.0–50.0) ^a^	29.7 (0.0–50.0)		
“A friendly demeanor of my family physician is more important to me than their knowledge level.”	164 (44.3)	159 (43.0)	47 (12.7)	χ^2^ = 4.184	0.123
	30.7 (0.0–50.0)	31.2 (0.0–50.0)	28.2 (0.0–48.9)		
“The proximity of a health institution to my home affects which institution I choose for my health problems.”	140 (37.8)	191 (51.7)	39 (10.5)	χ^2^ = 9.262	**0.010**
	32.8 (0.0–50.0) ^a^	29.7 (0.0–50.0)	28.1 (0.0–46.3) ^a^		
“For an illness, the more medications I take, the faster and easier I recover.”	270 (73.0)	69 (18.6)	31 (8.4)	χ^2^ = 4.914	0.086
	30.7 (0.0–50.0)	30.2 (0.0–50.0)	28.1 (0.0–50.0)		
“When visiting a family health center for an examination, I prefer to be examined only for the current complaint rather than having a full general examination.”	134 (36.2)	203 (54.9)	33 (8.9)	χ^2^ = 14.811	**0.001**
	31.8 (0.0–50.0) ^a^	30.2 (0.0–50.0) ^b^	24.5 (0.0–50.0) ^ab^		
“When choosing a family physician, I prefer more experienced physicians over younger ones.”	183 (49.5)	144 (38.9)	43 (11.6)	χ^2^ = 5.580	0.061
	31.8 (0.0–50.0)	30.2 (0.0–50.0)	28.1 (0.0–49.5)		

χ^2^: Kruskal–Wallis Test, ^ab^: Same letters express significant differences between groups. Bold indicates statistically significant values (*p* < 0.05).

**Table 3:** Results of the generalized linear model with the Turkish Health Literacy Scale-32 (THLS-32) score as the dependent variable.

	B	Std. Error	95% CI	*p*
Constant	33.700	2.943	27.932, 39.468	**<0.001**
Sex (male)	−2.739	1.312	−5.311, −0.167	**0.037**
Education (university graduate)	9.215	2.045	5.207, 13.223	**<0.001**
Education (vocational school graduate)	10.005	2.313	5.472, 14.538	**<0.001**
Education (high school graduate)	7.066	1.597	3.936, 10.196	**<0.001**
Occupation (student)	−3.239	2.771	−8.670, 2.192	0.242
Occupation (retired)	−2.117	2.851	−7.705, 3.471	0.458
Occupation (self-employed)	−0.690	1.810	−4.238, 2.858	0.703
Occupation (worker)	−3.961	1.962	−7.807, −0.115	**0.044**
Occupation (civil servant)	−1.129	1.976	−5.002, 2.744	0.568
Regular medication use (yes)	1.627	1.461	−1.237, 4.491	0.266
Age	−0.196	0.063	−0.319, −0.073	**0.002**
Marital status (married)	0.964	1.446	−1.870, 3.798	0.505
Marital status (widowed/divorced)	3.778	2.608	−1.334, 8.890	0.147

Reference categories: Sex = female; Education level = illiterate/primary/middle school graduate; Occupation = unemployed/housewife, Marital status = single. B: unstandardized regression coefficient; 95% CI calculated as B ± 1.96 × SE (Wald approximation). Bold indicates statistically significant values (*p* < 0.05).

## Data Availability

The data presented in this study are available upon request from the corresponding author due to ethical/privacy restrictions on participant data.

## Supplementary Materials

The following supporting information can be downloaded at: https://www.mdpi.com/article/10.3390/healthcare14183114/s1, Supplementary Material S1: Data collection questionnaire; Supplementary Material S2: Turkish Health Literacy Scale-32 (THLS-32)—English Translation; Supplementary Material S3: STROBE Statement—Checklist of Items for Cross-Sectional Studies.

## Abbreviations

Health Literacy	HL
Turkish Health Literacy Scale-32	THLS-32
European Health Literacy Survey	HLS-EU
Generalized Linear Model	GLM
