# Supplementary material for: Patient Expectations, Satisfaction and Perceptions of Specialization in Family Medicine: A Cross-Sectional Study from a Health Literacy Perspective in Türkiye
Source: Healthcare (Basel). 2026 Sep 21;14(18):3114. doi: 10.3390/healthcare14183114 (PMC13606649; doi:10.3390/healthcare14183114)
Supplement: Supplementary file 1 [file healthcare-14-03114-s001.zip › Suplementary Material S1_revised.pdf]

**Supplementary Material S1.**	Data collection questionnaire	**Disagree**	**Agree**	**Undecided**
Statement 1	"In Family Health Centers, only general practitioners (non-specialists) work as physicians."			
Statement 2	"A family medicine specialist becomes a specialist after one year of training."			
Statement 3	"It is important to me that my family physician is a family medicine specialist."			
Statement 4	"I believe I have sufficient knowledge about the authorities and competencies of family medicine specialists."			
Statement 5	"A family medicine specialist has the authority to issue medication reports for chronic diseases."			
Statement 6	"If I had to change my family physician one day, I would prefer a doctor who fulfills my requests most easily rather than the one I think is the most knowledgeable or skilled."			
Statement 7	"If I believe I will recover with antibiotics, I would not hesitate to tell my physician."			
Statement 8	"I would be pleased if my family physician does not refuse my request for antibiotics."			
Statement 9	"I am pleased if my family physician closely follows medical advancements."			
Statement 10	"I would take a medication prescribed by my family physician without hesitation."			
Statement 11	"Except for emergencies, I would not consider going to a hospital for health problems without my family physician's referral."			
Statement 12	"I prefer to be able to go directly to a training and research hospital, city hospital, or medical faculty hospital whenever I want."			
Statement 13	"Physicians working in hospitals are superior to those working in family health centers."			
Statement 14	A friendly demeanor of my family physician is more important to me than their knowledge level.			
Statement 15	"The proximity of a health institution to my home affects which institution I choose for my health problems."			
Statement 16	"For an illness, the more medications I take, the faster and easier I recover."			
Statement 17	"When visiting a family health center for an examination, I prefer to be examined only for the current complaint rather than having a full general examination."			
Statement 18	"When choosing a family physician, I prefer more experienced physicians over younger ones."			

Note: Statements are grouped into three domains as follows:

(A) Perceptions/Knowledge of the Family Medicine Specialization System: Statements 1, 2, 4, 5, 13.

(B) Expectations Regarding Family Physician Care: Statements 3, 6, 11, 12, 14, 15, 17, 18.

(C) Satisfaction-Related Beliefs and Behaviors: Statements 7, 8, 9, 10.

Statement 16 reflects a general health belief not specific to family medicine specialization and is therefore not classified under a specific domain.
