# Supplementary material for: Patient Expectations, Satisfaction and Perceptions of Specialization in Family Medicine: A Cross-Sectional Study from a Health Literacy Perspective in Türkiye
Source: Healthcare (Basel). 2026 Sep 21;14(18):3114. doi: 10.3390/healthcare14183114 (PMC13606649; doi:10.3390/healthcare14183114)
Supplement: Supplementary file 1 [file healthcare-14-03114-s001.zip › Supplementary_Material_S2_THLS-32.pdf]

Supplementary Material S2. Turkish Health Literacy Scale-32 (THLS-32) — English Translation

The following 32 items constitute the Turkish Health Literacy Scale-32 (THLS-32), originally developed and validated in Turkish by Okyay, P. and Abacıgil, F. (Eds.) in "Türkiye Sağlık Okuryazarlığı Ölçekleri Güvenilirlik ve Geçerlilik Çalışması" (Republic of Türkiye Ministry of Health Publication No. 1025, Ankara, 2016). The scale is reproduced here in unofficial English translation, for reference purposes only, to facilitate assessment of the study methodology; it is not intended as a validated English-language instrument. The original Turkish source is openly available at: https://acikders.ankara.edu.tr/pluginfile.php/114974/mod_resource/content/1/Sa%C4%9Fl%C4%B1k%20Okur%20Yazarl%C4%B1%C4%9F%C4%B1%20%C3%96l%C3%A7ekleri.pdf

Instructions to respondents: "If you were to rate the following from very easy to very difficult, how easy or difficult is it for you to do the following?" Each item is rated on a 4-point scale (Very Easy / Easy / Difficult / Very Difficult) plus a "Don't Know" option.

**No.**	**Item**	**Very Easy**	**Easy**	**Difficult**	**Very Difficult**	**Don't Know**
**1**	Finding out whether a health complaint you have is a symptom of an illness.					
**2**	Reading and understanding any written material (brochure, booklet, poster, etc.) related to a health complaint you have.					
**3**	Judging whether the advice of your family or friends is reliable when you have a health complaint.					
**4**	Finding out which doctor you should consult when you want to visit a health facility.					
**5**	Finding out how to make an appointment (e.g., booking a visit) when you want to go to a health facility.					
**6**	Making an appointment by phone or internet when you want to go to a health facility.					
**7**	Finding information about the treatments for illnesses that concern you.					
**8**	Understanding your doctor's explanations about your illness.					
**9**	Weighing the advantages and disadvantages of different treatment options recommended by your doctor.					
**10**	Using your medications as recommended by health professionals (doctor, pharmacist, etc.).					
**11**	Understanding the usage instructions included in the medication package.					
**12**	Deciding whether you need a second opinion from a different doctor.					
**13**	Understanding information about preparations before a test/examination (e.g., following a diet).					
**14**	Finding your way to the unit you need to reach in a hospital (e.g., laboratory, outpatient clinic).					
**15**	Deciding what to do in an emergency (e.g., accident, sudden health problem).					

No.	Item	Very Easy	Easy	Difficult	Very Difficult	Don't Know
**16**	Calling an ambulance when necessary.					
**17**	Undergoing regular health check-ups and follow-ups as recommended by your doctor.					
**18**	Finding information about conditions that could be harmful to your health, such as being overweight or having high blood pressure.					
**19**	Understanding health warnings about conditions that could be harmful to your health, such as being overweight or having high blood pressure.					
**20**	Finding information about how to cope with unhealthy behaviors such as smoking or insufficient physical activity.					
**21**	Understanding health warnings about unhealthy behaviors such as smoking or insufficient physical activity.					
**22**	Finding information about health screenings you should undergo based on your age, sex, and health status (e.g., breast screening for women, prostate-related screenings for men).					
**23**	Understanding information recommended for becoming healthier from sources such as the internet, newspapers, television, or radio.					
**24**	Deciding whether information recommended for becoming healthier from sources such as the internet, newspapers, television, or radio is reliable.					
**25**	Understanding information on food packaging that you think may affect your health.					
**26**	Evaluating the positive and negative features of your environment (home, street, neighborhood, etc.) that affect health.					
**27**	Finding information about what can be done to make your environment (home, street, neighborhood, etc.) healthier.					
**28**	Assessing which of your daily behaviors (such as exercising, eating healthily, not smoking) affect your health.					
**29**	Changing your lifestyle for your health (such as exercising, eating healthily, not smoking).					
**30**	Following a diet plan given in writing by a dietitian.					
**31**	Making recommendations to your family or friends about how to be healthier.					
**32**	Interpreting policy changes related to health.					

Raw item coding (as administered): 1 = Very Easy, 2 = Easy, 3 = Difficult, 4 = Very Difficult; "Don't Know" is coded separately. Reverse-scoring: prior to index calculation, raw codes are reverse-scored (1↔4, 2↔3) so that higher recoded values reflect greater ease, consistent with the scale's standard scoring procedure. Scoring: Index = (mean of reverse-scored item responses − 1) × (50/3), yielding a range of 0–50, where 0 = lowest HL and 50 = highest HL. Items answered as "Don't Know" are treated as missing and excluded from the calculation of the mean item score; participants with valid responses to fewer than 80% of items are excluded from index calculation. Classification: 0–25 = inadequate HL; >25–33 = problematic–limited HL; >33–42 = sufficient HL; >42–50 = excellent HL.
