# Supplementary material for: Patient Expectations, Satisfaction and Perceptions of Specialization in Family Medicine: A Cross-Sectional Study from a Health Literacy Perspective in Türkiye
Source: Healthcare (Basel). 2026 Sep 21;14(18):3114. doi: 10.3390/healthcare14183114 (PMC13606649; doi:10.3390/healthcare14183114)
Supplement: Supplementary file 1 [file healthcare-14-03114-s001.zip › Supplementary_Material_S3_STROBE.pdf]

Supplementary Material S3. STROBE Statement — Checklist of Items for Cross-Sectional Studies

This checklist follows the STROBE (Strengthening the Reporting of Observational Studies in Epidemiology) guideline for cross-sectional studies (von Elm, E.; Altman, D.G.; Egger, M.; Pocock, S.J.; Gøtzsche, P.C.; Vandenbroucke, J.P.; STROBE Initiative. The Strengthening the Reporting of Observational Studies in Epidemiology (STROBE) Statement: guidelines for reporting observational studies. Lancet 2007, 370, 1453–1457). Each item indicates where the corresponding information is reported in the manuscript. Where an item was not fully addressed, this is noted transparently with cross-reference to the relevant limitation.

**Item**	**STROBE Item**	**Recommendation**	**Reported in manuscript (section/location)**
**1a**	Title	Indicate the study's design with a commonly used term in the title.	Title: "...A Cross-Sectional Study from a Health Literacy Perspective in Türkiye." — study design explicitly stated.
**1b**	Abstract	Provide an informative and balanced summary in the abstract of what was done and what was found.	Abstract (Background/Objectives, Methods, Results, Conclusions).
**2**	Background/rationale	Explain the scientific background and rationale for the investigation being reported.	Introduction, paragraphs 1-4 (quality of care, gatekeeping, health literacy definition and impacts, gap in literature).
**3**	Objectives	State specific objectives, including any prespecified hypotheses.	Introduction, final paragraph: "This study aimed to assess patients' responses to a set of study-specific items on expectations, satisfaction, and perceptions..."
**4**	Study design	Present key elements of study design early in the paper.	Methods, "Study Design and Participants": described as single-center, prospective, cross-sectional survey.
**5**	Setting	Describe the setting, locations, and relevant dates, including periods of recruitment, exposure, follow-up, and data collection.	Methods, "Study Design and Participants" (single Family Health Center, Çifteler, rural district of Eskişehir Province) and "Data Collection Procedure" (data collected 15/03/2025–15/05/2025).
**6a**	Participants	Give the eligibility criteria, and the sources and methods of selection of participants.	Methods, "Inclusion and Exclusion Criteria"; sampling method described in "Study Design and Participants" (convenience sampling, consecutive approach in clinic waiting area).

Item	STROBE Item	Recommendation	Reported in manuscript (section/location)
**6b**	Participants (cross-sectional specific)	For matched studies, give matching criteria and number of exposed and unexposed. (Not applicable — no matching used in this cross-sectional design.)	Not applicable; no matched design was used.
**7**	Variables	Clearly define all outcomes, exposures, predictors, potential confounders, and effect modifiers.	Methods, "Data Collection Procedure" (sociodemographic variables, expectations/satisfaction/perceptions items) and "Scale Description and Assessment" (THLS-32 as the primary outcome/exposure variable).
**8**	Data sources/measurement	For each variable of interest, give sources of data and details of methods of assessment (measurement). Describe comparability of assessment methods if more than one group.	Methods, "Data Collection Procedure" and "Scale Description and Assessment" (mixed self-completion/face-to-face administration described; THLS-32 scoring procedure detailed).
**9**	Bias	Describe any efforts to address potential sources of bias.	Methods, "Data Collection Procedure" (face-to-face THLS-32 administration to reduce comprehension-related bias); Study Limitations (selection bias, response bias, non-response bias discussed).
**10**	Study size	Explain how the study size was arrived at.	Methods, "Sample Size Estimation" (Cochran's formula, population of 6,240 visits, minimum n=362).
**11**	Quantitative variables	Explain how quantitative variables were handled in the analyses. If applicable, describe which groupings were chosen and why.	Methods, "Scale Description and Assessment" (THLS-32 index calculation and categorization into 4 HL levels); "Statistical Analyses" (age treated continuously; education/occupation/marital status as categorical).
**12a**	Statistical methods (a)	Describe all statistical methods, including those used to control for confounding.	Methods, "Statistical Analyses" (Kruskal-Wallis, Mann-Whitney U, Spearman correlation, GLM for confounder adjustment).
**12b**	Statistical methods (b)	Describe any methods used to examine subgroups and interactions.	Not formally examined; noted as a limitation (item-level associations in Table 2 are unadjusted, exploratory, without subgroup/interaction

Item	STROBE Item	Recommendation	Reported in manuscript (section/location)
			testing) — Statistical Analyses and Study Limitations.
**12c**	Statistical methods (c)	Explain how missing data were addressed.	Methods, "Inclusion and Exclusion Criteria" (THLS-32 'don't know' responses >20% → excluded) and "Scale Description and Assessment" (item-level 'don't know' treated as missing).
**12d**	Statistical methods (d)	Cross-sectional studies: N/A (item applies to cohort sampling strategy / case-control matching).	Not applicable to this cross-sectional design.
**12e**	Statistical methods (e)	Describe any sensitivity analyses.	No formal sensitivity analyses (e.g., alternative model specifications) were conducted. Marital status was included as a covariate in the primary adjusted model (Table 3) rather than tested separately — Statistical Analyses.
**13a**	Participants (a)	Report numbers of individuals at each stage of the study — eligible, examined, confirmed eligible, included, completing follow-up, analyzed.	Methods, "Sample Size Estimation" and "Inclusion and Exclusion Criteria" report final analytic sample (n=370). The number of patients approached and declining participation was not systematically recorded — disclosed as a limitation in Study Limitations.
**13b**	Participants (b)	Give reasons for non-participation at each stage.	Not available — disclosed as a limitation (response rate could not be calculated) in Study Limitations.
**13c**	Participants (c)	Consider use of a flow diagram.	Not included, as detailed participant flow data (numbers approached/declined) were not systematically recorded during data collection; disclosed in Study Limitations.
**14a**	Descriptive data (a)	Give characteristics of study participants (e.g., demographic, clinical, social) and information on exposures and potential confounders.	Results, Table 1 (sociodemographic characteristics and unadjusted associations with THLS-32 score).
**14b**	Descriptive data (b)	Indicate the number of participants with missing data for each variable of interest.	Partially reported: THLS-32 'don't know'/exclusion threshold described in Methods; item-level missing-data

Item	STROBE Item	Recommendation	Reported in manuscript (section/location)
			counts for individual questionnaire items are not separately tabulated.
**14c**	Descriptive data (c)	Cohort studies: summarize follow-up time. Not applicable (cross-sectional design).	Not applicable to this cross-sectional design.
**15**	Outcome data	Cross-sectional studies: Report numbers of outcome events or summary measures.	Results, opening paragraph and Table 1 (THLS-32 mean score, HL category distribution, 60.3% inadequate/limited HL).
**16a**	Main results (a)	Give unadjusted estimates and, if applicable, confounder-adjusted estimates and their precision. Make clear which confounders were adjusted for and why they were included.	Results, Table 1 (unadjusted) and Table 3 (GLM-adjusted, with rationale for covariate selection described in Statistical Analyses).
**16b**	Main results (b)	Report category boundaries when continuous variables were categorized.	Methods, "Scale Description and Assessment" (THLS-32 index category boundaries: 0–25, >25–33, >33–42, >42–50).
**16c**	Main results (c)	If relevant, consider translating estimates of relative risk into absolute risk for a meaningful time period.	Not applicable — no relative risk estimates were computed in this cross-sectional analysis.
**17**	Other analyses	Report other analyses done — e.g., analyses of subgroups and interactions, and sensitivity analyses.	Results/Discussion: item-level (Table 2) exploratory comparisons and marital status sensitivity check within the GLM; explicitly labeled as exploratory/hypothesis-generating in Statistical Analyses and Study Limitations.
**18**	Key results	Summarize key results with reference to study objectives.	Discussion, opening paragraphs and Conclusion.
**19**	Limitations	Discuss limitations of the study, taking into account sources of potential bias or imprecision. Discuss both direction and magnitude of any potential bias.	Study Limitations (cross-sectional design, mixed administration format, single-center/selection bias, socioeconomic status bias, non-response rate, unadjusted item-level associations and multiple testing, absence of a validated satisfaction instrument, THLS-32 internal consistency not re-verified).
**20**	Interpretation	Give a cautious overall interpretation considering objectives, limitations, multiplicity of analyses, results from similar studies, and other relevant evidence.	Discussion (throughout); causal/behavioral language qualified relative to the cross-sectional design (e.g., antibiotic-seeking and

Item	STROBE Item	Recommendation	Reported in manuscript (section/location)
			examination-preference findings framed as expressed attitudes, not observed behavior).
**21**	Generalisability	Discuss the generalisability (external validity) of the study results.	Study Limitations (generalizability limited to care-seeking populations in similar geographic/institutional/rural contexts; socioeconomic representativeness noted).
**22**	Funding	Give the source of funding and the role of the funders for the present study and, if applicable, for the original study on which the present article is based.	End matter: "Funding: This research received no external funding."
